# SDAM: A dual attention mechanism for high-quality fusion of infrared and visible images

**DOI:** 10.1371/journal.pone.0308885

**Published:** 2024-09-24

**Authors:** Jun Hu, Xiaocen Zhu, Kai Niu

**Affiliations:** Changchun University of Science and Technology, ChangChun, JiLin, China; Tongji University, CHINA

## Abstract

Image fusion of infrared and visible images to obtain high-quality fusion images with prominent infrared targets has important applications in various engineering fields. However, current fusion processes encounter problems such as unclear texture details and imbalanced infrared targets and texture detailed information, which lead to information loss. To address these issues, this paper proposes a method for infrared and visible image fusion based on a specific dual-attention mechanism (SDAM). This method employs an end-to-end network structure, which includes the design of channel attention and spatial attention mechanisms. Through these mechanisms, the method can fully exploit the texture details in the visible images while preserving the salient information in the infrared images. Additionally, an optimized loss function is designed to combine content loss, edge loss, and structure loss to achieve better fusion effects. This approach can fully utilize the texture detailed information of visible images and prominent information in infrared images, while maintaining better brightness and contrast, which improves the visual effect of fusion images. Through conducted ablation experiments and comparative evaluations on public datasets, our research findings demonstrate that the SDAM method exhibits superior performance in both subjective and objective assessments compared to the current state-of-the-art fusion methods.

## Introduction

Image fusion is a technique that combines images from different sensors, spectral ranges, or time points to create a more comprehensive and accurate image [1–3]. The fusion technique of infrared and visible light image has been widely applied in various fields, including military, aerospace, firefighting and rescue, medical, and environmental monitoring. Infrared and visible image fusion technology aims to achieve higher information content and richer texture details than either image alone. Infrared images can capture the thermal characteristics of targets, making them highly effective for target recognition in low-light or nighttime environments. However, infrared images lack texture information, making it difficult to describe details of targets. Visible images, on the other hand, can provide more texture and color information, but their recognition performance is limited in complex environments such as low-light or smoke. Therefore, fusing the two types of images can fully leverage their respective strengths and improve the ability to recognize, detect, and track targets.

Traditional pixel-level image fusion algorithms usually adopt pixel-level fusion strategies, such as weighted averaging or max/min operations on the pixels of two images [4, 5]. These methods include multiscale transforms [6, 7], principal component analysis [8, 9], sparse representation [10, 11], saliency region extraction [12] and other hybrid methods [13–15]. This method is simple and easy to understand, but there are problems with information redundancy and complex fusion strategies. In addition, traditional methods often do not consider the correlation and mutual influence between different features, and lack effective extraction and representation of target features.

Although fusion networks based on autoencoders have achieved good fusion performance [16–18], the need to manually set fusion rules within the entire fusion framework limits the algorithm’s flexibility and generalization ability. The fusion networks have not been trained on a large dataset of infrared and visible light, and the encoders and decoders still have shortcomings in feature extraction and reconstruction. Infrared and visible light image fusion models based on Generative Adversarial Networks [19, 20] mainly focus on improvements and optimizations in the structure and number of generators and discriminators. However, the details of visible light in the generated fusion images are not sufficiently comprehensive. Increasing the number of generators and discriminators makes the entire training process more complex, and the generator of the Generative Adversarial Network changes some details of the original image, leading to a severe halo phenomenon in the final fused image. In end-to-end fusion networks [21, 22], fusion rules can be learned, and the trained fusion network can be quickly embedded into other advanced visual tasks. However, end-to-end fusion networks require training on a large-scale dataset of infrared and visible light images, and the design of the loss function is relatively complex.

However, deep learning-based methods also face several challenges and issues. Firstly, these methods typically require a large amount of data for training to achieve good performance. This can limit the feasibility of the methods in situations where data is limited, and significant time and resources are required to collect and annotate the data. Secondly, deep learning-based methods also have certain requirements in terms of computational resources. Deep neural networks often have a large number of parameters and complex computational processes, necessitating powerful computing capabilities for training and inference. This poses challenges in resource-constrained environments or devices. Additionally, there is a balance issue between brightness and texture details when fusing infrared and visible light images. Finding a balance point is crucial to fully leverage their respective strengths and compensate for their limitations.

To address these issues, researchers continue to propose new methods to optimize the performance and applications of infrared and visible image fusion technology. This paper proposes a method for infrared and visible image fusion based on a specific dual-attention mechanism, which fully utilizes the detailed information of visible images while retaining prominent information in infrared images. By employing carefully designed dual attention mechanisms, we can ensure that the fused images possess clear and rich texture information, resulting in optimal visual effects.

In summary, the contributions of this research are as threefold:

1. We designed an end-to-end fusion network structure that achieves image fusion without the need for complex fusion rules, significantly simplifying the process and enhancing the model’s efficiency and adaptability. By incorporating skip connections between layers, our network facilitates information reuse between shallow and deep features. This design not only enhances the effectiveness of feature transmission but also significantly improves the quality of the fused images, resulting in superior detail and overall performance.
2. We developed a specific dual attention mechanism, consisting of a channel attention module and a spatial attention module. The channel attention module helps the model automatically select the most relevant and distinctive feature channels, further extracting and emphasizing key features in infrared and visible light images, thereby enhancing the semantic information. The spatial attention module enables the model to focus on key areas within the image, enhancing texture details and improving the brightness and contrast of the fused image. By combining these two attention mechanisms, our network achieves superior performance in multiple aspects, significantly improving the quality of the fused images.
3. We formulated a well-designed loss function that jointly optimizes the network using a combination of content loss, edge loss, and structural loss. This multi-faceted loss function ensures that the fused images are improved in several key areas: content loss preserves the main information and structure of the source images, edge loss enhances edge information for clearer contours, and structural loss retains and enhances the overall structure information, leading to better grayscale distribution and texture details. Our experiments demonstrate that the network trained with this loss function produces fused images with more even grayscale distribution and clearer contour texture information.

## Related work

Currently, most researchers are mainly focusing on studying image fusion algorithms based on deep learning. Image fusion methods based on deep learning have strong robustness and generalization capabilities and are widely used to solve problems related to computer vision and image processing. In this section, we briefly review and discuss the works on image fusion based on CNN and the related research on attention mechanisms.

### CNN-based methods

In the early stages of research on infrared and visible light image fusion, commonly used traditional methods included simple weighted averaging and fusion methods based on wavelet transforms. These methods are simple but often fail to fully utilize the characteristics of different band images, thus limiting the improvement of fusion effects. Indeed, with the continuous advancements in deep learning technology, researchers have begun to explore its application in the field of infrared and visible light image fusion. As an illustration, Li et al. [23] introduced a methodology that leverages ResNet-50 as a pre-trained network for feature extraction. Subsequently, they employed zero-phase component analysis (ZCA) to compute fusion weight maps for infrared and visible images, followed by weighted averaging to produce the fusion image. Although this approach combines pre-trained networks with traditional methods for image fusion, the fusion efficiency achieved is relatively low.

In contrast, Ma et al. [24] presented a GAN-based approach for infrared and visible image fusion termed GANMcC. This method employs a generative adversarial training framework, utilizing a generator and a discriminator, to continuously improve the fusion performance of the network and generate fused images with rich source image information. The generator serves as the core component of the network, responsible for fusing the infrared and visible light images and generating a high-quality fused image. The discriminator acts as an auxiliary component, used to evaluate the authenticity of the fused images generated by the generator.

Furthermore, Ma et al. [25] roposed a network called STDFusion for infrared and visible image fusion, based on the principle of salient object detection. The design of this network aims to preserve the thermal object information from the infrared image and the texture structure from the visible light image. During the training phase of the network, it requires the provision of mask information for salient objects as auxiliary inputs to guide the network in learning the location and importance of the objects. However, this also adds complexity to the training process and may result in limited generalization ability of the network when dealing with unseen data.

Zhang et al. [26] proposed a generic image fusion framework based on Convolutional Neural Networks (IFCNN). IFCNN consists of three modules: a feature extraction module, a feature fusion module, and an image reconstruction module. Salient image features are extracted from multiple input images using two convolutional layers. The convolutional features of multiple input images are fused according to appropriate fusion rules based on the type of input images. The fused features are then reconstructed through two convolutional layers to produce an information-fused image. Li et al. [27] introduced a novel end-to-end fusion network structure for infrared and visible light image fusion (RFN-Nest). RFN is used to fuse multimodal deep features extracted at each scale. While shallow features retain more detail information, deep features contain semantic information, which is crucial for reconstructing salient features. The fused image is reconstructed through a decoder network based on nested connections, fully utilizing the multi-scale structure of features. Jian et al. [28] proposed an auto-encoder network with residual blocks (SEDRFuse) for fusing infrared and visible light images. During the training phase, the SEDRFuse network is trained to create a fixed feature extractor. In the fusion phase, the trained feature extractor is used to extract intermediate and compensatory features, which are generated by the residual blocks of the input source images and the first two convolutional layers, respectively. This method designed an attention-based feature fusion strategy to merge intermediate features, addressing the issue of partial feature information loss due to network deepening.

Li et al. [29] proposed a novel Salient Object Segmentation (SOS) network to effectively highlight infrared foreground thermal information by segmenting the foreground information from infrared images, and based on the saliency mask and source image A new IMV-F fusion strategy is proposed. This strategy utilizes spatial and channel attention to fuse foreground and background respectively, highlighting foreground information and retaining rich visible light background details. Zhou et al. [30] designed the Information Quantity Discrimination (IQD) module to generate appropriate fusion weights for each semantic object, and proposed a dual discriminator method to achieve high semantic consistency fusion images, but while retaining There is still room for improvement in terms of semantic information.

Methods such as FAFusion [31]explore the use of low- and high-frequency information in fusion of infrared and visible light images. FAFusion uses frequency-aware and residual multi-scale feature extraction modules to significantly improve the texture details and target saliency of fused images. Experiments on TNO, MSRS and M3FD data sets have verified its effectiveness. IAIFNet [32] solves the challenges in fusion of infrared and visible images in low-light environments by introducing an illumination enhancement network and a salient object perception module, outperforming existing methods on the LLVIP dataset. In addition, this method is also combined with an adaptive differential fusion module to achieve efficient image fusion and significant performance improvement in low-light environments.

The above CNN-based image fusion methods mainly modify the fusion framework and formulate loss functions to constrain the fusion process. By exploring the balance relationship between infrared target brightness and visible texture features, the quality of fusion images can be further improved. Among them, deep learning technology can help extract higher-level feature information, better utilize the characteristics of different band images, and improve the quality and accuracy of fusion images. Simultaneously, through the constraint and optimization of the image fusion process, it is possible to achieve further enhancements in fusion efficiency and generalization ability. This, in turn, allows for better fulfillment of the requirements in diverse application scenarios.

### Attention mechanism

Attention mechanism is a technique used to improve model performance, which can help the model better utilize key information in the input data, thus achieving more accurate and efficient prediction and inference [33, 34]. Attention mechanism is commonly used to solve tasks such as image classification, object detection, and image segmentation. T The attention mechanism introduces a weight vector into the model, indicating which parts of the input data are most important for the model’s output. Indeed, in deep learning-based fusion models, there are two common implementations of the attention mechanism: channel attention and spatial attention. Both mechanisms enable the model to better utilize crucial information from the input data, resulting in more accurate and efficient predictions and inferences.

The channel attention mechanism is a technique used to improve model performance, which can help the model better utilize the information in each channel of the input data, thus achieving more accurate and efficient prediction and inference [35, 36]. Channel attention involves introducing a weight vector to indicate the importance of each channel in the model’s output. Specifically, the channel attention mechanism is implemented by computing weights for each channel and then performing a weighted summation of the input data across all channels to obtain the final output. The channel attention mechanism was proposed by Hu et al. [37] in 2018 and utilizes the Squeeze-and-Excitation (SE) module to achieve adaptive channel weighting. The latest channel attention mechanisms further optimize this method, for example, by using the Channel Attention Module (CAM) [38] to process the channel information in the input data and using the Global Context Block (GCB) to process global information [39].

The spatial attention mechanism facilitates better utilization of information from different positions in the input data, leading to more accurate and efficient predictions and inferences [40, 41]. Spatial attention involves introducing a weight matrix into the model, indicating the importance of different positions in the input data for the model’s output. This mechanism was initially proposed by Woo et al. in 2018 and applied in the field of deep learning, utilizing the Spatial Attention Module (SAM) [37] for adaptive spatial weighting. The latest spatial attention mechanisms further optimize this method, for example, by using the Position Attention Module (PAM) [42] to process the relationships between different positions in the input data and using the Non-Local Block (NLB) to process global information [43].

Channel attention and spatial attention mechanisms are two commonly used attention mechanisms in deep learning, which can help the model better utilize key information in the input data, thus achieving more accurate and efficient prediction and inference. The latest research results show that channel attention and spatial attention mechanisms perform very well in handling complex scenes and large-scale datasets. Researchers will continue to explore new methods and techniques to improve the channel attention and spatial attention mechanisms. These improvements may include more precise weight calculation methods, more efficient model architecture designs, and more effective training algorithms, among others.

## Proposed method

In this section, we first provide a detailed description of the design methodology and relevant parameters of the network structure, in conjunction with the network architecture diagram. Next, we offer a detailed account of the structures of the designed channel attention module and spatial attention module. Finally, we describe the details of the loss functions used for training the network.

### Architecture of the fusion network

**Fig 1:**
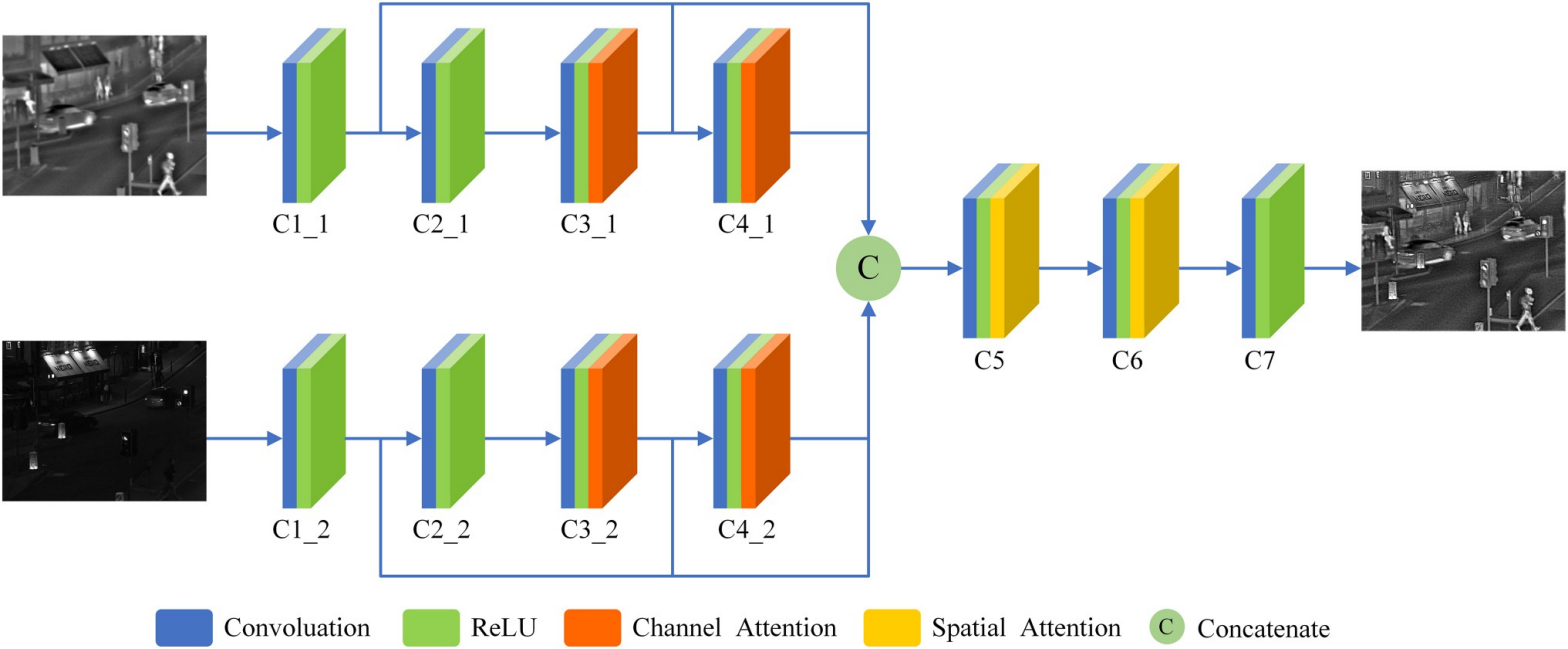
SDAM network structure.

(1) The proposed SDAM adopts an end-to-end network architecture, utilizing seven convolutional layers, channel attention modules, and spatial attention modules to extract features from the infrared and visible light images. In this architecture, the first four convolutional layers perform feature extraction on the infrared and visible light images separately. The outputs of the first, third, and fourth convolutional layers from both branches are then concatenated and used as input to the fifth convolutional layer. The overall structure diagram is illustrated in Fig 1.

**Fig 2:**
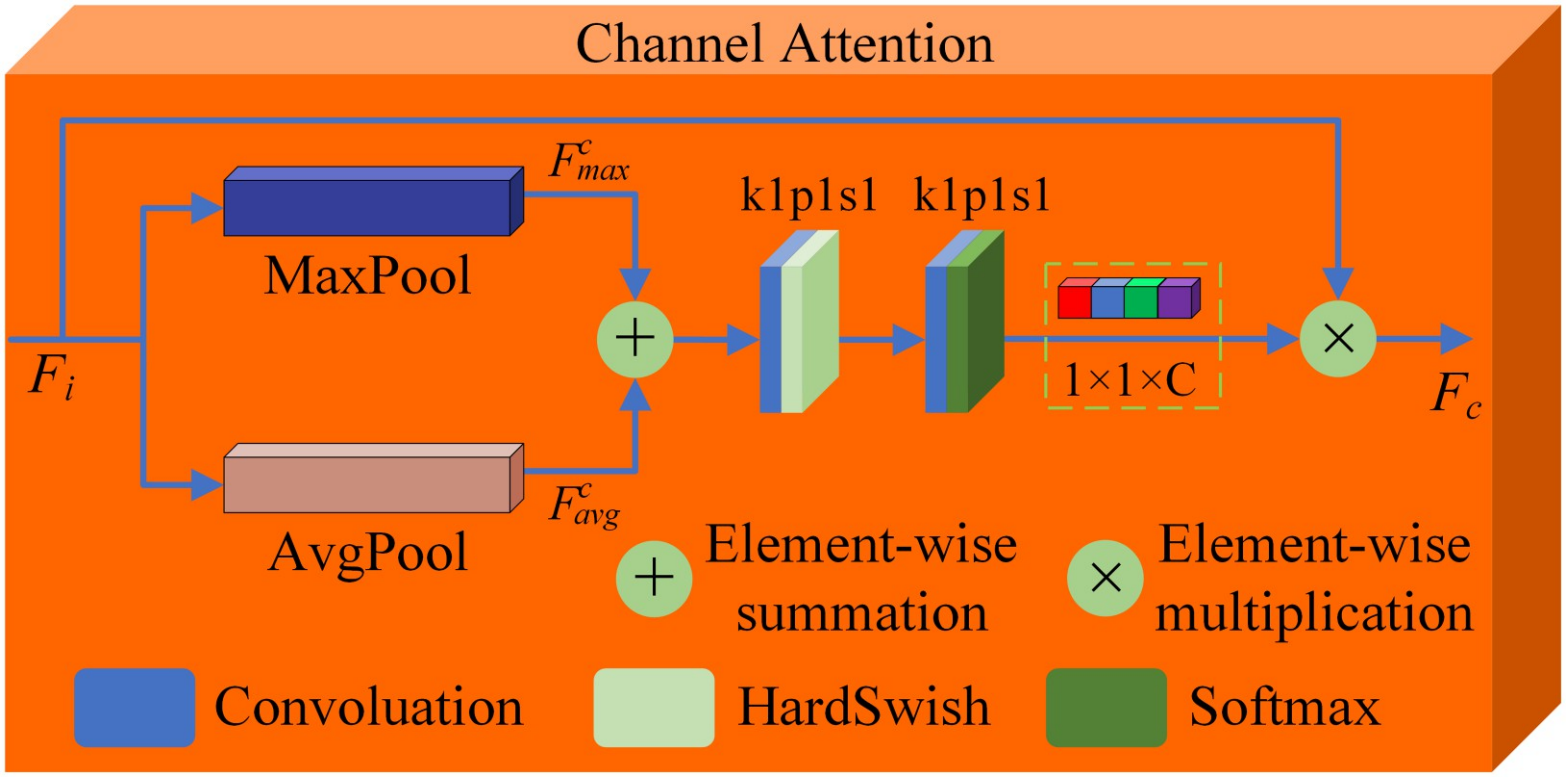
Structure of the channel attention module.

**Fig 3:**
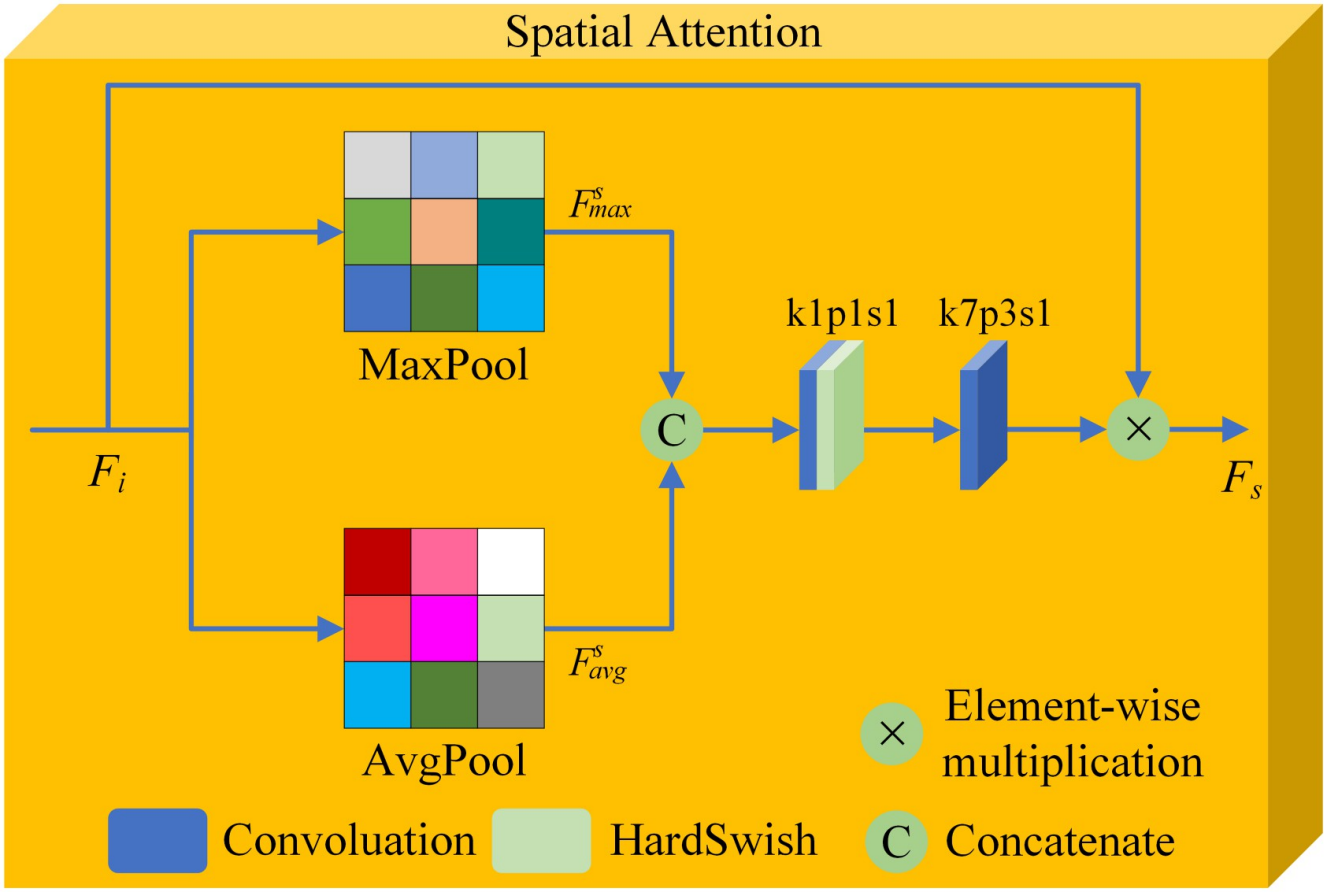
Structure of the spatial attention module.

The specific network parameters are shown in Table 1. In the SDAM network, the activation function used for the C7 layer is Tanh. As for the C1-C6 layers, the rectified linear unit (ReLU) is employed as the activation function. By carefully selecting these activation functions, the SDAM network can better adjust the grayscale distribution of the output image to align with the characteristics of human visual observation. This helps improve the quality and visual effects of the fused image, enhancing the perception and understanding of the targets.To obtain more feature information from the input images, the first convolutional layer in SDAM network uses a 5×5 convolution kernel. The advantage of using channel attention module in C3 and C4 is that it can adaptively predict potential key channels and improve the ability to capture global information. The purpose of adding spatial attention module in C5 and C6 is to mimic the human visual system, which can efficiently analyze and understand complex scenes, extract important feature information for fusion, and obtain a fused image with better visual effect. The structures of the channel attention module and spatial attention module are shown in Figs 2 and 3, respectively.

**Table 1:** SDAM network parameters.

Layer	Kernel	Stride	Channel (input)	Channel (output)	Padding	Activation
C1_1	5×5	1	1	16	SAME	ReLU
C1_2	5×5	1	1	16	SAME	ReLU
C2_1	3×3	1	16	32	SAME	ReLU
C2_2	3×3	1	16	32	SAME	ReLU
C3_1	3×3	1	32	64	SAME	ReLU
C3_2	3×3	1	32	64	SAME	ReLU
C4_1	3×3	1	64	64	SAME	ReLU
C4_2	3×3	1	64	64	SAME	ReLU
C5	3×3	1	224	64	SAME	ReLU
C6	3×3	1	64	16	SAME	ReLU
C7	3×3	1	16	1	SAME	Tanh

To avoid losing the details and texture information from the source images during the fusion process, the SDAM fusion network decides not to employ downsampling operations. By maintaining the same resolution as the source images, the network can better preserve the details and structure of the original images, thereby achieving more accurate and refined fusion results.

(2) Channel Attention Module: The advantage of the channel attention module is that it can adaptively select the importance of input features, thereby improving the robustness and generalization ability of the model. In addition, it can also reduce the number of parameters of the model and improve the computational efficiency of the model. By calculating the importance weight of each channel, the proportion of channel output is dynamically adjusted. This enables the model to adaptively select the importance of input features. To obtain global features for each channel, max pooling and average pooling are commonly used dimensionality reduction operations that can be applied to the feature maps of each channel. In the channel attention module we designed, $F_{Max}^{c}$ and $F_{Avg}^{c}$ can be obtained by computing the maximum and average pooling of the feature maps for each channel. The outputs of the third and fourth convolution blocks in the processing branches for infrared and visible light images are used as inputs to the channel attention module. In this process, maximum pooling and average pooling operations are employed to compute $F_{\text{max}}^{c}$ and $F_{\text{avg}}^{c}$, respectively. These two intermediate quantities represent the channel attention module’s response to different features. Subsequently, the outputs of the fifth and sixth convolution blocks are used as inputs to the spatial attention module. Similarly, maximum pooling and average pooling operations are utilized to obtain $F_{\text{max}}^{s}$ and $F_{\text{avg}}^{s}$, which reflect the spatial attention module’s emphasis on different spatial locations. Subsequently, sum the results and perform two convolution operations followed by a softmax computation to obtain the channel attention map, with the dimensions of the channel attention map being 1 × 1 × *C*. The calculation formula of *M*_*c*_ is as follows,

$$\begin{array}{c} M_{c}=Softmax(Conv^{1\times 1}(\sigma (Conv^{1\times 1}(Sum(F_{Max}^{c},F_{Avg}^{c}))))) \end{array}$$ (1)

where *Conv*^1×1^ represents the use of 1×1 convolution, *σ* represents the HardSwish activation function [44], and *Sum* represents element-wise summation. Assuming that the input feature map is *F*_*i*_, the feature map output by the channel attention module is *F*_*c*_, and the calculation formula of *F*_*c*_ is as follows,

$$\begin{array}{c} F_{c}=M_{c}\left(F_{i}\right)⊗F_{i} \end{array}$$ (2)

where ⊗ represents element-wise multiplication.

(3) Spatial Attention Module: The spatial attention module can adaptively select important parts of the input data and help the model focus on the important regions and positions in the input data. The spatial attention module dynamically adjusts the proportion of input data by calculating the importance weight of each spatial position. The calculation process of the spatial attention module is relatively simple, making it easier to explain the model’s decision-making process. In the spatial attention module, spatial features such as edges, corners, etc., denoted as $F_{Max}^{s}$ and $F_{Avg}^{s}$. The outputs of the fifth and sixth convolution blocks are used as inputs to the spatial attention module. Similarly, maximum pooling and average pooling operations are utilized to obtain $F_{\text{max}}^{s}$ and $F_{\text{avg}}^{s}$, which reflect the spatial attention module’s emphasis on different spatial locations.The convolutional layer calculates the weight of each spatial position by learning the relationship between different spatial positions. Finally, the spatial attention map *M*_*s*_ is obtained. The calculation formula of *M*_*s*_ is as follows,

$$\begin{array}{c} M_{s}=Conv^{7\times 7}(\sigma (Conv^{1\times 1}(Concat(F_{Max}^{s},F_{Avg}^{s})))) \end{array}$$ (3)

where *Conv*^7×7^ denotes the use of a 7 × 7 convolution, *Conv*^1×1^ denotes the use of a 1 × 1 convolution, *σ* denotes the HardSwish activation function, and *Concat* denotes concatenation. Let *F*_*i*_ be the input feature map, and let *F*_*s*_ be the feature map output by the channel attention module. *F*_*s*_ is calculated as follows,

$$\begin{array}{c} F_{s}=M_{s}\left(F_{i}\right)⊗F_{i} \end{array}$$ (4)

where ⊗ represents element-wise multiplication.

### Loss function

The choice of loss function plays a crucial role in optimizing the image fusion model and obtaining superior fusion results. By utilizing pixel-level loss functions, the model can learn image features at a fine-grained level, leading to the generation of more detailed and realistic fusion results. These loss functions are widely employed in infrared and visible light image fusion tasks, providing effective guidance for model learning and optimization. Pixel-level loss functions help the model learn pixel-level details, such as the hotspots in the infrared image and the color information in the visible light image. By minimizing the pixel-level differences, the model can better capture the correspondence between the two images, resulting in more refined fusion outcomes.

The total loss function *L*_*total*_ designed during the training process consists of three parts: content loss *L*_*content*_, edge loss *L*_*edge*_, and structure loss *L*_*structure*_. The design of the structure loss function is inspired by some of the content in the SSIM [45] loss function. The total loss can be expressed as follows,

$$\begin{array}{c} L_{total}=\alpha L_{content}+\beta L_{edge}+\gamma L_{structure} \end{array}$$ (5)

where the parameters *α*, *β*, and *γ* are scaling factors that control the proportions of the three loss functions. Their purpose is to balance the magnitudes of the three loss terms, thereby accelerating model training and obtaining improved fusion results. The calculation methods of *L*_*content*_, *L*_*edge*_, and *L*_*structure*_ are as follows,

$$\begin{array}{c} L_{content}=\frac{1}{HW}\sum_{i}^{H}\sum_{j}^{W}(\|I_{fused}^{\left(i,j\right)}-max(I_{ir}^{\left(i,j\right)},I_{vis}^{\left(i,j\right)}){\|}_{F}^{2}) \end{array}$$ (6)

$$\begin{array}{c} L_{edge}=\frac{1}{HW}\sum_{i}^{H}\sum_{j}^{W}(\|\nabla I_{fused}^{\left(i,j\right)}-max(\nabla I_{ir}^{\left(i,j\right)},\nabla I_{vis}^{\left(i,j\right)}){\|}_{F}^{2}) \end{array}$$ (7)

$$\begin{array}{c} L_{structure}=\frac{\sigma_{I_{fused}I_{ir}}+c_{1}}{\sigma_{I_{fused}}\sigma_{I_{ir}}+c_{1}}+\frac{\sigma_{I_{fused}I_{vis}}+c_{2}}{\sigma_{I_{fused}}\sigma_{I_{vis}}+c_{2}} \end{array}$$ (8)

where *H* and *W* represent the height and width of the image, respectively. |⋅|*F* denotes the Frobenius norm of a matrix. The function *max*(⋅) outputs the maximum value, where a larger value implies that more salient information is retained. *I*_*fused*_ represents the fused image obtained from the network output, while *I*_*ir*_ and *I*_*vis*_ represent the input infrared and visible images, respectively. The symbol ∇ denotes the gradient calculation operator. The symbol *σ* represents the standard deviation. To prevent division by zero during the computation, the constants *c*_1_ and *c*_2_ are set to non-zero values.

To summarize, the SDAM network achieves optimal pixel distribution, rich edge details, and clear texture structure in the final fusion image by jointly constraining and optimizing the content loss, edge loss, and structural loss.

## Experimental analysis

In this section, we first provide a detailed overview of the experimental setup. Next, we briefly describe the datasets used for training and testing purposes. Subsequently, we conduct various ablation experiments to validate the rationality and effectiveness of the designed network structure. Lastly, we compare our proposed method with other state-of-the-art fusion methods on two different test datasets.

### Experimental details

The fusion model SDAM, designed in our work, was trained and tested on a hardware configuration consisting of an Intel(R) Core(TM) i9-13900KF processor, 64.0 GB RAM, and an NVIDIA GeForce RTX 4090 GPU. The computer’s software operating system of choice was Windows 11.

The SDAM fusion model was trained and optimized within the provided hardware and software environment. The Adam optimizer was utilized, with an initial learning rate set to 1 × 10^−4^, *β*_1_ set to 0.9, *β*_2_ set to 0.999, and *ε* set to 1 × 10^−8^. A minibatch size of 16 was employed. The SDAM model consisted of 100 epochs (iteration cycles), with adjustments made to the learning rate at the 50th and 80th epochs, reducing it to 0.1 from its original value.

During the testing process, we compared the trained SDAM model with the currently top 5 fusion methods based on deep learning frameworks, which are GANMcC [24], IFCNN [26], SEDRFuse [28], RFN-Nest [27], STDFusion [25], MrFDDGAN [30] and SOSMaskFuse [29]. To ensure the fairness of the testing experiments, we used the author’s open-source original code and relevant parameter settings in the paper to conduct experiments and obtain the best fusion performance.

### Dataset

**Fig 4:**
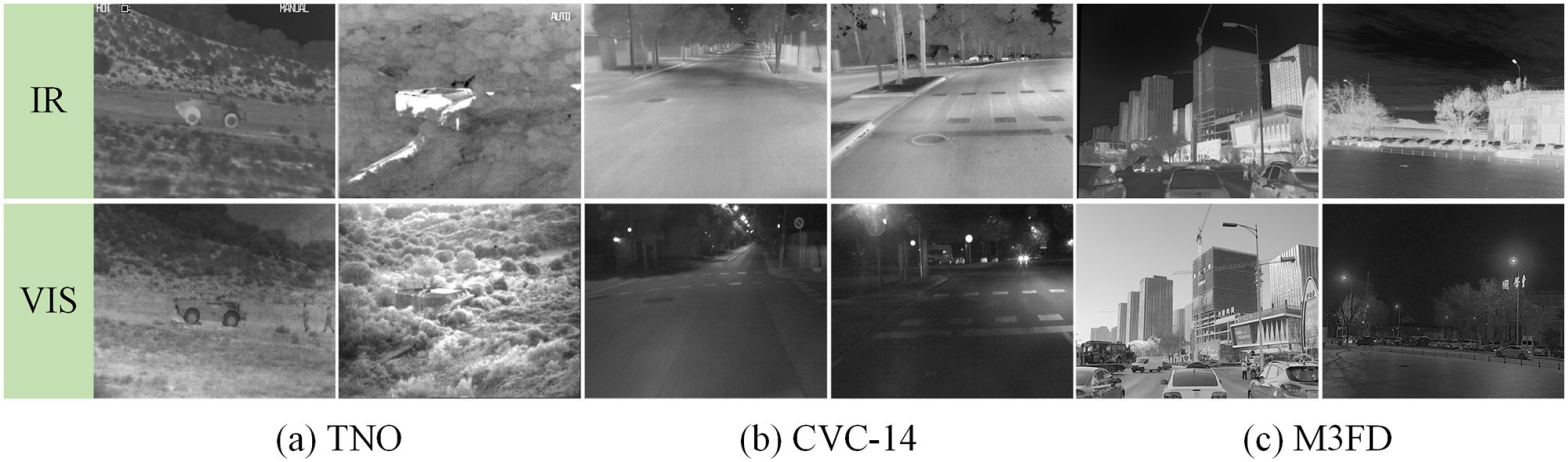
Partial examples of the dataset.

We utilized a large dataset of registered infrared and visible image pairs to train the SDAM model. A total of 11,615 pairs images were selected from commonly used image fusion datasets, namely TNO [46], CVC-14 [47], and M3FD [48] These images have undergone preprocessing and registration, fulfilling the requirements for training the SDAM model. Fig 4 illustrates sample infrared and visible images from the three datasets.

### Evaluation metrics

For evaluating the performance of the fusion network, both subjective and objective evaluations can be considered. Subjective evaluation is based on human visual characteristics and focuses on the visual quality of the fused images. Evaluators assess the quality of the fused images based on their subjective perception. In subjective evaluation, the fused images should strive to retain texture details, ensuring that the details of the objects remain visible after fusion. Additionally, the grayscale distribution of the fused image should align with human visual observation, making it more perceptually consistent in terms of brightness and contrast, thus improving the subjective evaluation scores. Nonetheless, relying solely on subjective evaluation is inadequate to provide a comprehensive assessment of the fusion network’s performance and is inherently unreliable.

To more fairly and objectively evaluate the performance of the fusion network, researchers have proposed various image evaluation metrics. We selected six evaluation metrics from different dimensions to test the images output by the fusion network. The six evaluation metrics are spatial frequency (SF) [49], edge intensity (EI) [50], contrast (CO) [51], information entropy (EN) [52], average gradient (AG) [53], and visual information fidelity (VIF) [54].

(1) Spatial Frequency (SF). Computing the gradient of an image can be used to reflect the level of detail and texture clarity in the fused image. Spatial frequency is primarily composed of the row frequency (RF) and column frequency (CF). The calculation formula for SF is as follows,

$$\begin{array}{c} SF=\sqrt{RF^{2}+CF^{2}} \end{array}$$ (9)

$$\begin{array}{c} RF=\sqrt{\frac{1}{M\times N}\sum_{i=1}^{M}\sum_{j=2}^{N}{\left[P(i,j)-P(i,j-1)\right]}^{2}} \end{array}$$ (10)

$$\begin{array}{c} CF=\sqrt{\frac{1}{M\times N}\sum_{i=2}^{M}\sum_{j=1}^{N}{\left[P(i,j)-P(i-1,j)\right]}^{2}} \end{array}$$ (11)

where *P*(*i*, *j*) represents the fused image with size *M* × *N*. When the SF value is larger, it signifies that the fused image performs better in preserving edge and texture information. These details are essential for the human visual system as they provide more information about object shape, structure, and surface features.

(2) Edge intensity (EI). Edge intensity refers to the degree of pixel grayscale value variation between the target and background regions. When calculating edge intensity, there is no need to reference an image. The calculation process of EI can be represented as follows,

$$\begin{array}{c} EI=\frac{\sqrt{\sum_{i=1}^{M}\sum_{j=1}^{N}s_{x}{\left(i,j\right)}^{2}+s_{y}{\left(i,j\right)}^{2}}}{M*N} \end{array}$$ (12)

$$\begin{array}{c} s_{x}=P*h_{x},s_{y}=P*h_{y} \end{array}$$ (13)

where *M*, *N* represent the width and height of the fused result image, and the results of the *Sobel* operator applied in the *x* and *y* directions are denoted as *h*_*x*_ and *h*_*y*_, respectively. The convolution results of the *Sobel* operator are represented as *s*_*x*_ and *s*_*y*_.

(3) Contrast (CO). Contrast is the ratio of black to white in an image, which means the gradient level from black to white. The larger the ratio, the more gradient levels from black to white, resulting in richer color representation. Contrast has a crucial impact on visual effects. Generally speaking, the higher the contrast, the clearer and more prominent the image, and the more vivid and bright the colors. However, if the contrast is too low, the grayscale values of the entire image may be too concentrated. The calculation process of CO can be represented as follows,

$$\begin{array}{c} CO=\sum_{\delta }\delta {\left(i,j\right)}^{2}P_{\delta }\left(i,j\right) \end{array}$$ (14)

where *δ*(*i*, *j*) = |*i* − *j*| represents the absolute value of the grayscale difference between adjacent pixels, and *P*_*δ*_(*i*, *j*) is the probability distribution of pixels with a grayscale difference of *δ* between adjacent pixels.

(4) Information entropy (EN). EN is a statistical feature used to measure the average information content in an image. It provides a measurement of the information distribution within the image, which is used to assess the complexity and diversity of the image. The calculation process of EN can be represented as follows,

$$\begin{array}{c} EN=\sum_{i=0}^{255}-P_{F}\left(i\right)\;{\text{log}}_{2}(P_{F}\left(i\right)) \end{array}$$ (15)

where *P*_*F*_(*i*) represents the statistical probability of the grayscale histogram of the fused infrared and visible image. The larger the value obtained by entropy calculation, the better it is, as a larger value contains more information, resulting in more details being retained in the fused image.

(5) Average Gradient (AG). The AG can independently evaluate the quality of the fused infrared and visible image. AG quantifies the level of edge information and detail variations within the image. The calculation process of AG can be represented as follows,

$$\begin{array}{c} AG=\frac{1}{MN}\sum_{i=1}^{M}\sum_{j=1}^{N}\sqrt{\frac{\nabla P_{x}^{2}\left(i,j\right)+\nabla P_{y}^{2}\left(i,j\right)}{2}} \end{array}$$ (16)

where ∇*P*_*x*_(*i*, *j*) = *P*(*i*, *j*) − *P*(*i* + 1, *j*) and ∇*P*_*y*_(*i*, *j*) = *P*(*i*, *j*) − *P*(*i*, *j* + 1). When the average gradient value of the fused image is larger, it can be considered as an indication of better performance of the fusion algorithm. This implies that the algorithm effectively preserves the details from the original images and produces fused results with good clarity.

(6) Visual Information Fidelity (VIF). VIF is a metric used to measure the fidelity of information in the fused image, similar to the perceptual ability of the human visual system. It assesses the extent to which the original information is preserved in the fused image. The calculation process of VIF can be represented as follows,

$$\begin{array}{c} VIF=\frac{\sum_{k\in \;\text{subband}\;}\sum_{b}I(C_{b},F_{b})}{\sum_{k\in \;\text{subband}\;}\sum_{b}I(C_{b},E_{b})}=\frac{\sum_{k}\sum_{b}{\text{log}}_{2}(1+\frac{g_{k,b}^{2}s_{k,b}^{2}C_{u}}{(\sigma_{V_{k,b}}^{2}+\sigma_{N}^{2})I})}{\sum_{k}\sum_{b}{\text{log}}_{2}(1+\frac{s_{k,b}^{2}C_{u}}{\sigma_{N}^{2}I})} \end{array}$$ (17)

where *k* and *b* stand for sub-band and block index, respectively; *g*_*k*,*b*_ is the scalar gain field in the *b*th block at the *k*th sub-band, and *s*_*k*,*b*_ and *C*_*U*_ are defined correspondingly. It is evident that *g*_*k*,*b*_ and *s*_*k*,*b*_ are generalized definitions of *g*_*i*_ and *s*_*i*_ when considering multiple sub-bands.

### Comparative experiment

**Fig 5:**
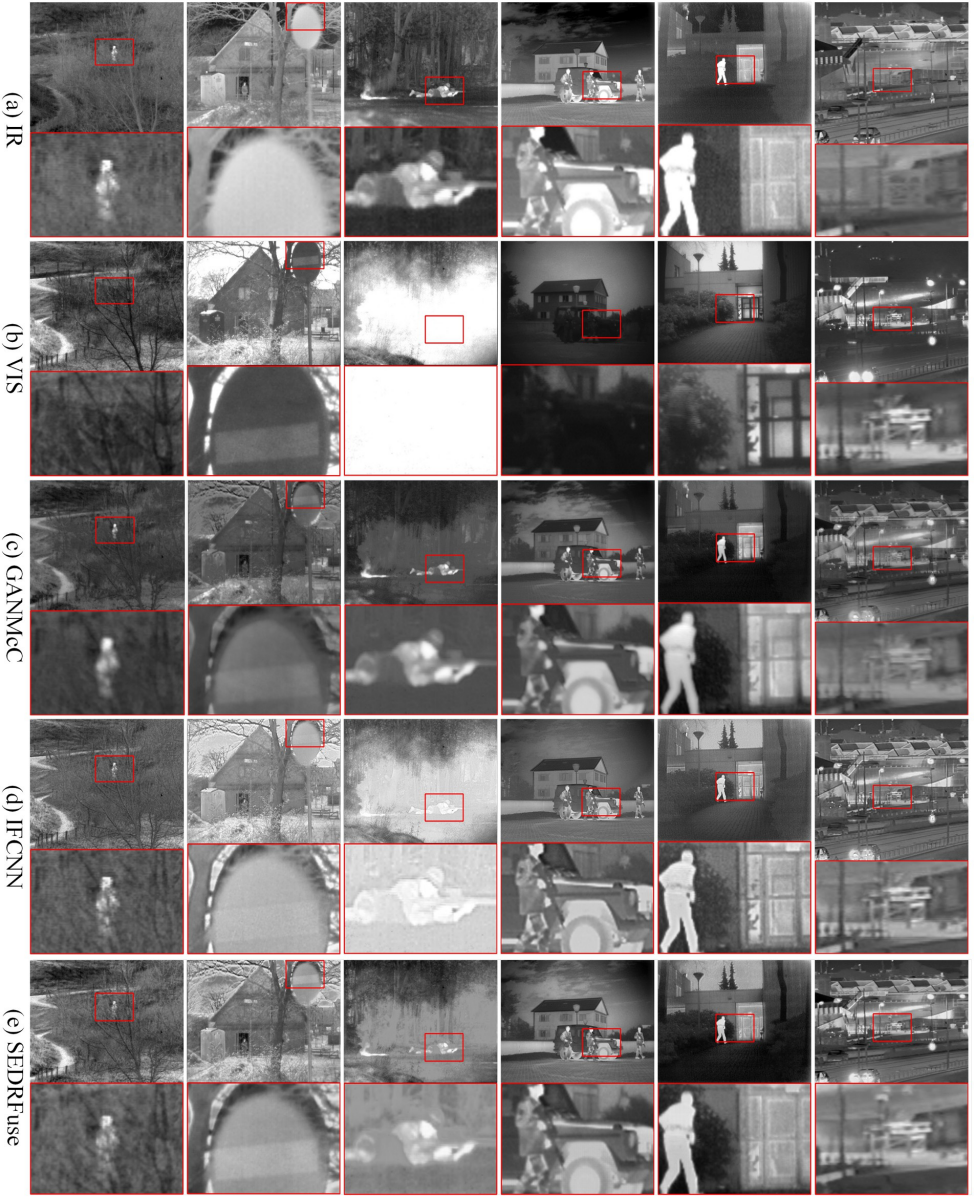
A qualitative comparison of SDAM with five state-of-the-art fusion methods on six typical pairs of infrared and visible images in the TNO dataset. From top to bottom, they are: (a) the infrared image, (b) the visible image, (c) the fusion results of GANMcC, (d) the fusion results of IFCNN, (e) fusion results of SEDRFuse.

**Fig 6:**
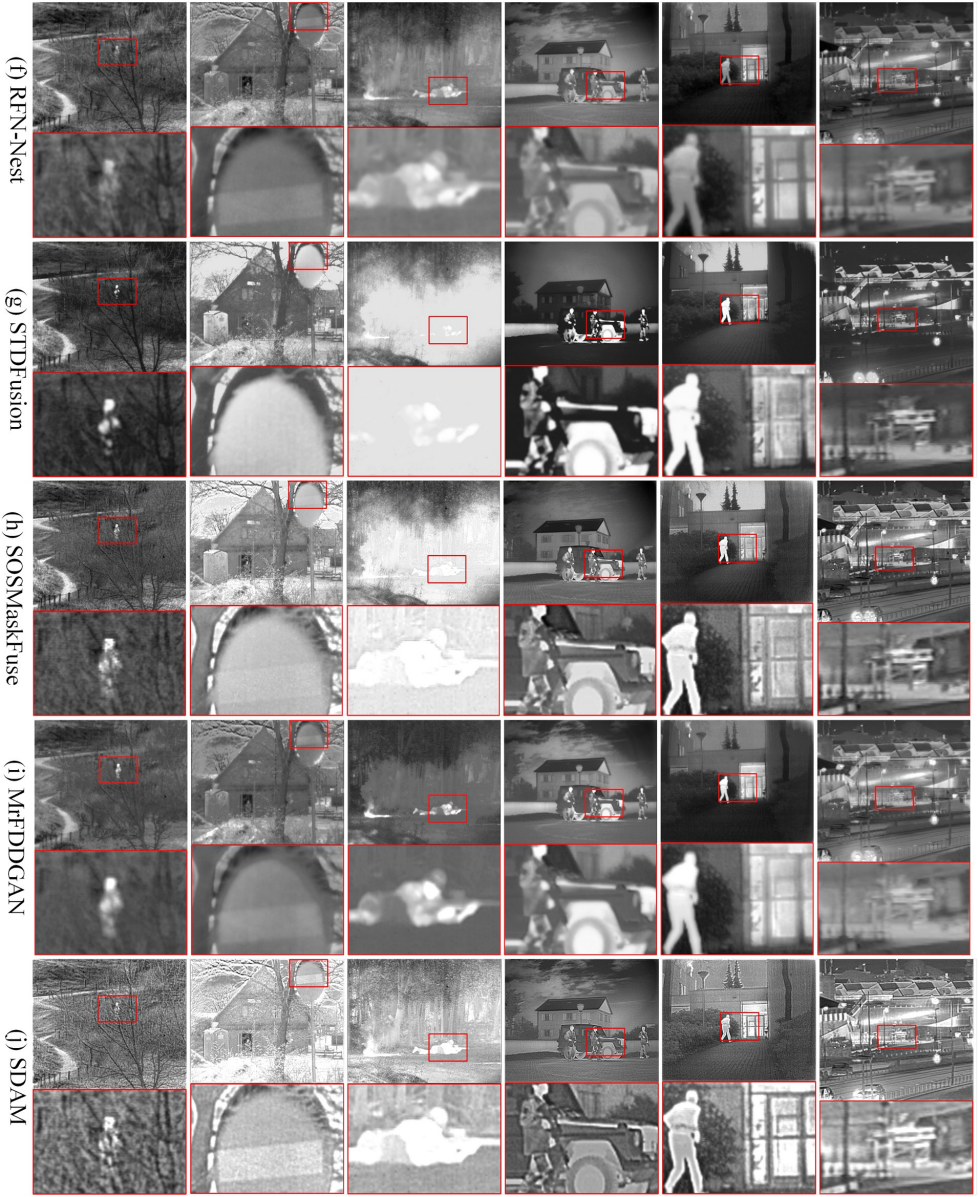
Qualitative comparison of SDAM and 5 state-of-the-art fusion methods on 6 typical pairs of infrared and visible images. From top to bottom: (f) fusion results of RFN-Nest, (g) fusion results of STDFusion, (h) fusion results of MrFDDGAN, (i) fusion results of SOSMaskFuse, (j) fusion results of SDAM.

(1) TNO dataset. To test the six fusion methods, we selected 30 pairs of infrared and visible images from the TNO dataset. These image pairs will be used to evaluate the performance and effectiveness of the fusion methods in different scenarios. The 30 image pairs were not involved in the training of the network. As shown in Figs 5 and 6, we selected six typical scenes from the test results of the six fusion methods for demonstration purposes. In the fused results, we zoomed in on the key regions and highlighted them in red to emphasize their importance. By zooming in on the key regions, we can carefully observe and analyze the details in the fusion results. This helps us gain a better understanding of how the fusion algorithms handle key regions and evaluate their performance in extracting and preserving critical information.

The fusion results of GANMcC and STDFusion exhibit noticeable lack of clarity, with blurred contours of pedestrians and vehicles. The visible light information in traffic signs is not adequately captured, resulting in a lack of display of the traffic sign information in the fusion results. This observation reveals that GANMcC and STDFusion fail to fully capture the background details in the visible image during the fusion process. In contrast, the fusion results obtained by IFCNN demonstrate high clarity, but may suffer from overexposure issues in certain scenes. Additionally, the defogging capability during the fusion process is weak, leading to the loss of texture details of pedestrians. The fusion results of SEDRFuse and RFN-Nest are adversely affected by significant noise interference, resulting in poor visual effects, low contrast, and blurred object contours.

The fusion results of MrFDDGAN demonstrate good preservation of textures and details, particularly excelling in complex scenes. However, it may introduce slight color distortions in some cases, especially in the color reproduction of traffic signs, sometimes not as vivid as the original visible light images. MrFDDGAN still performs excellently in overall fusion quality, providing a more balanced visual effect. SOSMaskFuse, in the process of fusing infrared and visible light images, places special emphasis on the effective extraction and synthesis of information, making the fusion results visually closer to real scenes. Compared to other fusion methods, SOSMaskFuse shows superior performance in maintaining natural colors and contrast of images, especially in the presentation of traffic signs and pedestrian details, demonstrating higher clarity and accuracy.

From the images, it can be observed that the fusion results of SDAM exhibit good visual effects, with a grayscale distribution that is more in line with human visual perception. In these six typical scenes, SDAM successfully captures the contour information of pedestrians and vehicles, presenting rich and clear features. By zooming in on the observed regions, we can clearly see the pedestrian and traffic sign information in the fusion images. This indicates that SDAM accurately preserves the details of these important targets in the infrared image, allowing them to be displayed in the fusion results. This is highly beneficial for tasks such as pedestrian recognition, traffic sign identification, and scene understanding.

Table 2 shows the average metric values of six different fusion methods on the TNO dataset. The maximum value of each metric is highlighted in bold and red. SDAM achieves the best performance in SF, EI, CO, AG, and VIF metrics. As observed from Figs 5 and 6, the fusion result of SDAM contains sufficient texture and contour information, which results in better visual effects for the human eye. Thus, it achieves the optimal values in SF, EI, CO, and AG quantitative evaluation metrics. SOSMaskFuse and MrFDDGAN closely follow in terms of contrast and average gradient, indicating that these methods are also highly effective in enhancing image details and contrast. In contrast, GANMcC and RFN-Nest score lower across all evaluation metrics, indicating a need for improvement in preserving image details and enhancing contrast. IFCNN and STDFusion perform well on multiple metrics, particularly in contrast and average gradient.

**Table 2:** Quantitative results on the TNO dataset. The maximum value is highlighted in red and bold.

Method	SF	EI	CO	EN	AG	VIF
GANMcC	3.2578	21.8421	13.6647	6.6292	2.1144	0.6205
IFCNN	5.5954	37.5521	53.3272	6.6404	3.8079	0.8937
SEDRFuse	4.8986	34.8105	35.2428	6.9174	3.4023	0.7138
RFN-Nest	3.2547	24.3657	13.4157	6.8893	2.2709	0.6817
STDFusion	5.4162	37.2809	54.7389	6.8275	3.7226	0.8371
MrFDDGAN	6.1137	39.4628	61.9804	6.9359	4.2285	0.8876
SOSMaskFuse	6.2643	42.4762	62.0174	**6.9381**	4.8764	0.9027
SDAM	**8.0318**	**64.9442**	**76.0674**	6.8467	**6.6295**	**0.9162**

SDAM effectively combines the thermal target information from the infrared image with the background texture information from the visible image in an efficient manner. By preserving the salient information of the thermal targets, the fusion results can accurately capture the thermal features of the objects, providing images with enhanced discriminability. Simultaneously, by leveraging the rich background texture information from the visible light image, the fusion results can better restore the true details and textures of the scene. As a result, SDAM achieves the optimal value in the VIF quantitative evaluation metric.

**Fig 7:**
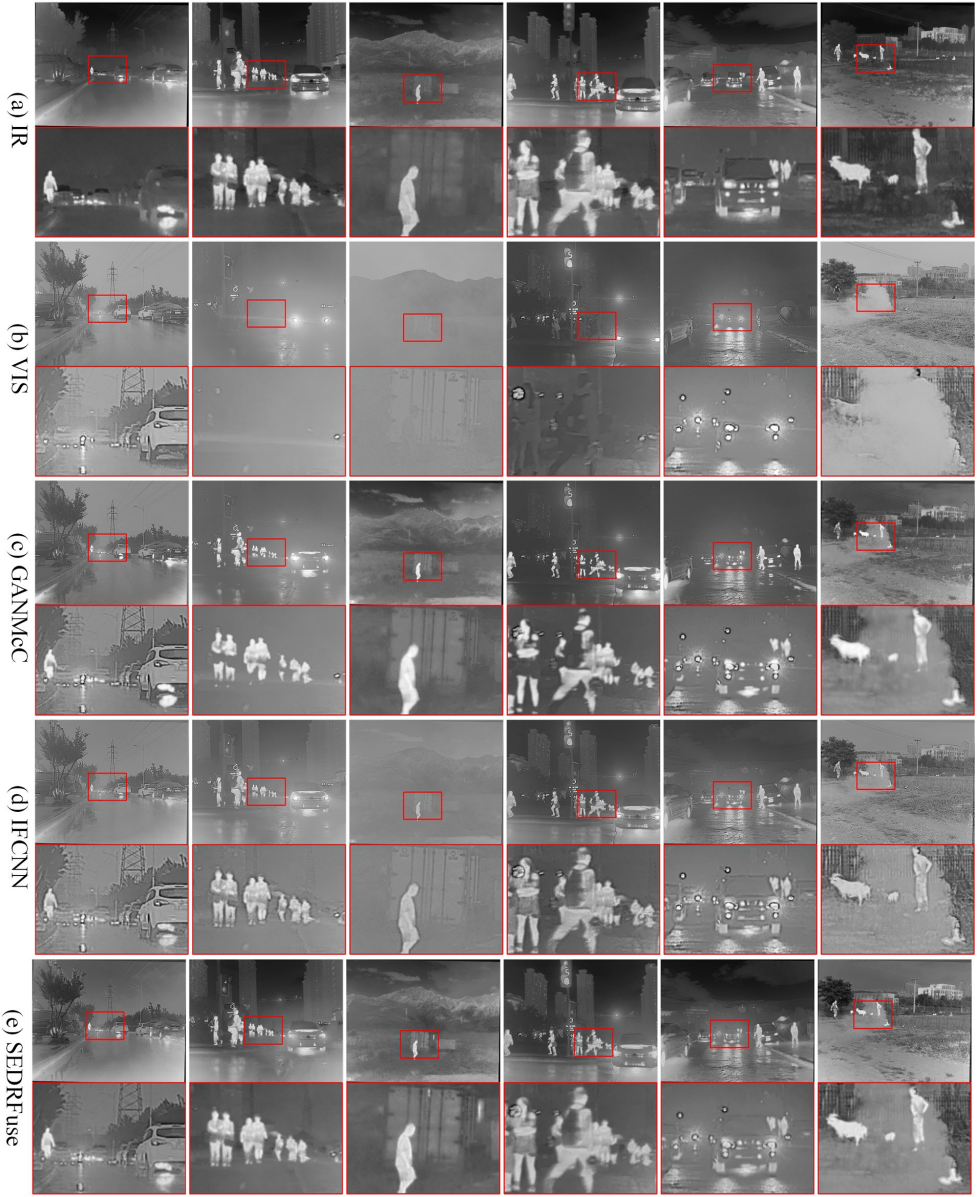
Qualitative comparison of SDAM with 5 state-of-the-art fusion methods on 6 typical infrared and visible image pairs in the M3FD dataset. From top to bottom: (a) the infrared image, (b) the visible image, (c) the fusion results of GANMcC, (d) the fusion results of IFCNN, (e) fusion results of SEDRFuse.

**Fig 8:**
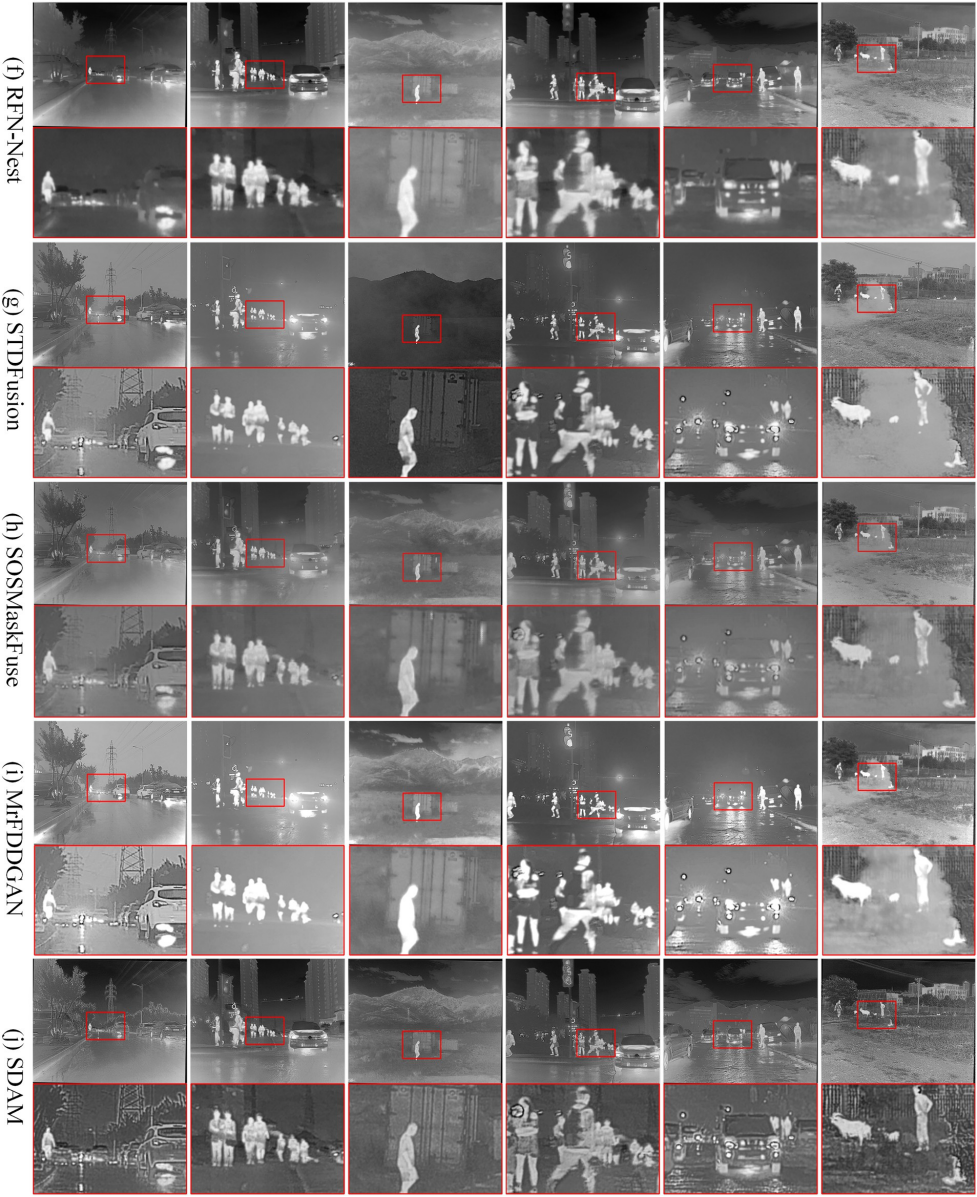
Qualitative comparison of SDAM and 5 state-of-the-art fusion methods on 6 typical pairs of infrared and visible images. From top to bottom: (f) fusion results of RFN-Nest, (g) fusion results of STDFusion, (h) fusion results of MrFDDGAN, (i) fusion results of SOSMaskFuse, (j) fusion results of SDAM.

(2) M3FD dataset. We selected 50 pairs of infrared and visible images that were not used in the network training to validate the performance of the SDAM method in fusion. Such testing helps evaluate the generalization ability and practical application value of the SDAM method. We selected the results of 6 fusion methods under 6 typical scenes for display, as shown in Figs 7 and 8.

The fusion outcomes obtained from GANMcC and RFN-Nest exhibit a lack of pedestrian details and a relatively blurry background, indicating insufficient preservation of texture information from the visible image. Moreover, in foggy scenes, both of these fusion methods exhibit limited defogging capabilities and are unable to effectively enhance the salient information captured in the infrared image. Conversely, the fusion outcome achieved through IFCNN exhibits prominent noise and overall blurriness, resulting in a subpar visual effect. Evidently, the visual quality of IFCNN is subpar. The SEDRFuse and STDFusion methods still fail to achieve satisfactory fusion results, as they do not effectively preserve the detailed texture information from the visible image and lose some important salient features.

During the fusion process, MrFDDGAN exhibits good preservation of texture information and remarkable feature enhancement capabilities. Compared to GANMcC and RFN-Nest, MrFDDGAN is more effective in revealing details of pedestrians and vehicles, and also demonstrates a stronger dehazing effect in foggy scenes. SOSMaskFuse, on the other hand, displays its superior fusion performance in multiple aspects. SOSMaskFuse not only clearly presents the contours of pedestrians, vehicles, and buildings but also effectively enhances significant information in the scene, such as heat sources and bright areas, making the fused image visually richer and more three-dimensional. Additionally, SOSMaskFuse also stands out in its dehazing ability, effectively improving overall visual clarity while maintaining the natural colors of the image.

In contrast, the SDAM method showcases clear details of pedestrians, vehicles, and buildings in the fusion results. By adequately preserving the detailed texture information from the visible light image and the salient features from the infrared image, the SDAM method exhibits clear target details in the fusion results, achieving optimal visual effects and defogging capabilities. Among the mentioned methods, only the fusion results of SDAM achieve the best visual quality, presenting clear contours, textures, and scene information of the targets. This is highly advantageous for subsequent image processing tasks.

According to the data in Table 3, the SDAM fusion method achieves optimal values in five quantitative evaluation metrics, namely SF, EI, CO, EN, and AG, for the M3FD dataset. It obtains a suboptimal value in terms of VIF. From Figs 7 and 8, it can be observed that the fusion results of SDAM retain a significant amount of edge and texture information, leading to optimal values in SF, EI, and AG. In the six typical scenes of the M3FD dataset, the SDAM fusion method demonstrates high clarity, rich detail information, and excellent visual effects.

**Table 3:** Quantitative results on the M3FD dataset. The maximum value is highlighted in red and bold.

Method	SF	EI	CO	EN	AG	VIF
GANMcC	5.5612	36.5476	52.4017	6.4825	3.3271	0.7135
IFCNN	5.6388	45.3541	98.3445	6.5918	4.7142	**0.9126**
SEDRFuse	4.8438	34.2926	56.3338	6.8739	3.2926	0.7629
RFN-Nest	3.2206	21.3981	19.5217	7.2279	2.9867	0.7152
STDFusion	5.9728	43.9535	108.0441	6.4263	4.4552	0.8691
MrFDDGAN	6.1572	47.2294	106.4692	7.0946	5.3926	0.9135
SOSMaskFuse	6.1958	49.5347	109.3857	7.1408	5.5837	0.9286
SDAM	**6.7269**	**62.7615**	**113.7069**	**7.2521**	**5.9312**	0.9086

SOSMaskFuse and MrFDDGAN exhibit excellent performance on the CO and AG metrics, demonstrating their effectiveness in enhancing image contrast and details. SOSMaskFuse slightly outperforms MrFDDGAN in EI and VIF, indicating its slightly superior capability in preserving edge information and enhancing visual quality compared to MrFDDGAN. STDFusion and IFCNN also perform well on the CO and AG metrics, but score lower on EI and VIF compared to SDAM, SOSMaskFuse, and MrFDDGAN, suggesting these methods have certain limitations in preserving edge information and enhancing visual quality. SEDRFuse shows a balanced performance across all evaluation metrics, indicating that SEDRFuse is a robust fusion method capable of providing satisfactory results in different aspects, yet lacks competitiveness in specific performance metrics. RFN-Nest and GANMcC score lower across all evaluation metrics, particularly underperforming in CO and AG, indicating these methods have weaker capabilities in enhancing image contrast and details, necessitating further improvements and optimizations. Therefore, the SDAM fusion method exhibits outstanding fusion performance on the M3FD dataset, effectively preserving the features of source images. The high values of these quantitative evaluation metrics further validate the exceptional performance of the SDAM method in fusion.

### Ablation experiment

The loss function, channel attention, and spatial attention are the three main components of the fusion approach suggested in this study. We carried out ablation experiments and identified the ideal settings for the loss function in order to further examine how these three designs affected the functionality of the fusion network. We also verified the SDAM network’s channel and spatial attention efficacy. These experiments were conducted on the TNO dataset, and quantitative evaluation metrics were calculated for each experimental group to perform comparative analysis. To ensure the fairness of the experiments, we only modified the parameters that required comparison, while keeping all other parameters and settings unchanged.

**Fig 9:**
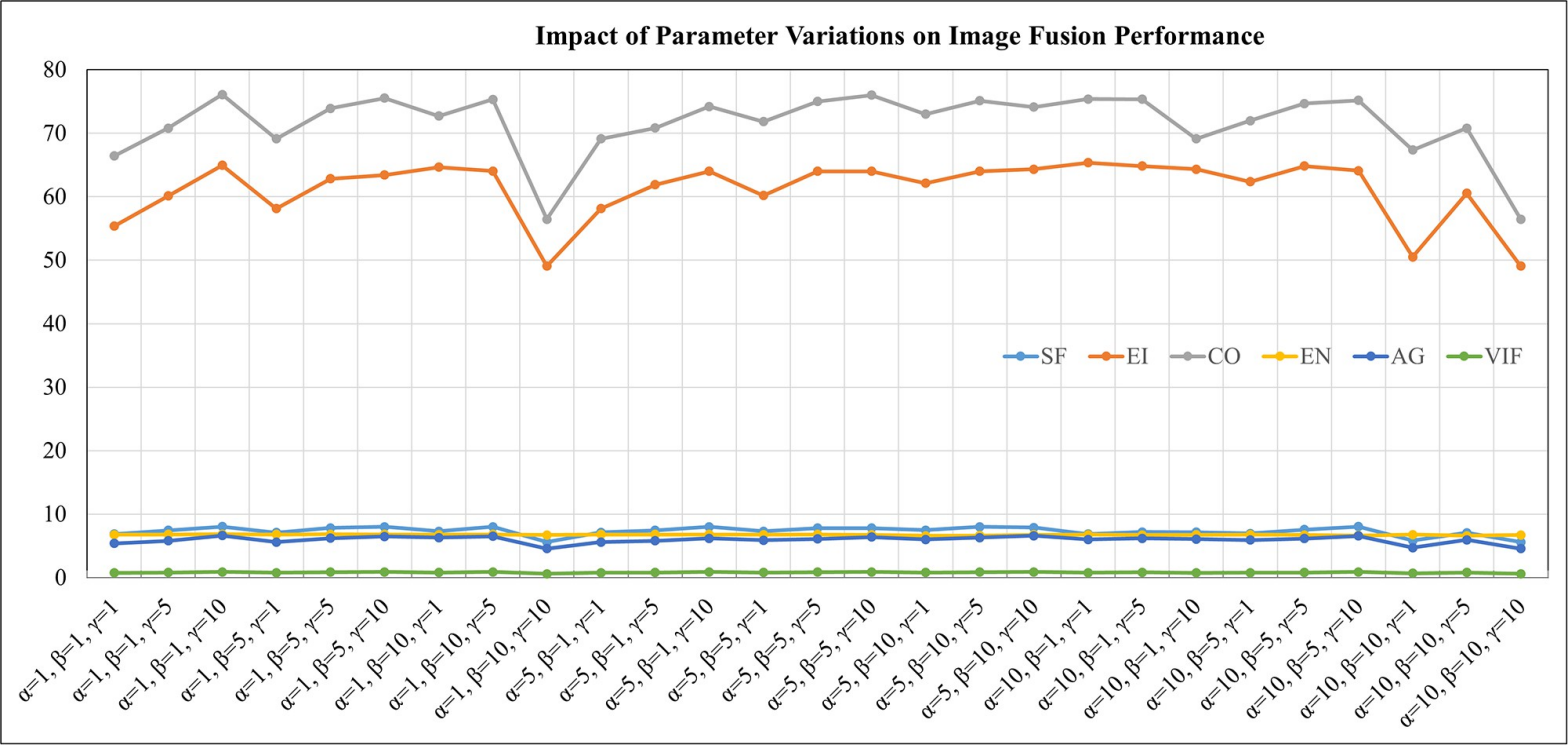
Impact of parameter variations on image fusion performance.

(1) Parameter Setting Experiment: In this paper, we propose a fusion method that optimizes the network using content loss, edge loss, and structural loss, balanced by the parameters *α*, *β*, and *γ*. To further verify the impact of parameter settings on the fusion performance, we conducted 27 experiments using different combinations of *α*, *β*, and *γ*. The detailed results are shown in the Fig 9.

**Fig 10:**
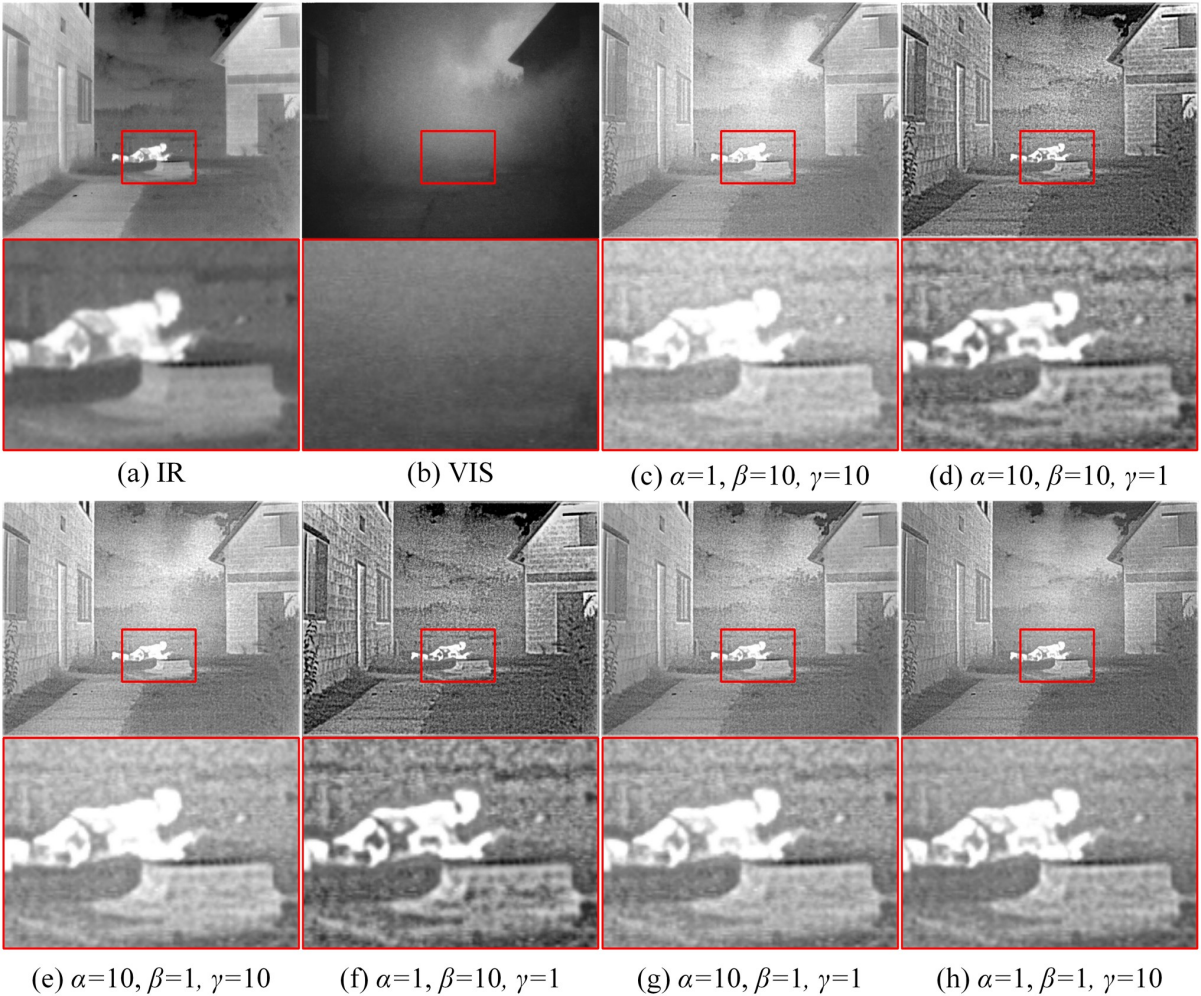
Representative results of ablation experiments under different parameters. (a) Infrared image, (b) visible image, (c) *α* = 1, *β* = 10, *γ* = 10, (d) *α* = 10, *β* = 10, *γ* = 1, (e) *α* = 10, *β* = 1, *γ* = 10, (f) *α* = 1, *β* = 10, *γ* = 1, (g) *α* = 10, *β* = 1, *γ* = 1, (h) *α* = 1, *β* = 1, *γ* = 10.

When *α* = 1, *β* = 10, *γ* = 10 and *α* = 10, *β* = 10, *γ* = 1, the overall fusion results are excessively bright but with lower contrast, losing some texture details and lacking sufficient edge contour information. When *α* = 10, *β* = 1, *γ* = 10, as shown in Fig 10, the house edges are overexposed, and the magnified area shows significant noise, leading to poor overall visual effect. When *α* = 1, *β* = 10, *γ* = 1 and *α* = 10, *β* = 1, *γ* = 1, although the image contrast is improved, the edge information of the clouds in the sky and the trees is unclear, with noticeable noise in the image. In summary, appropriate parameter selection is crucial for obtaining high-quality fusion results. Overall, when *α* = 1, *β* = 1, *γ* = 10, the fusion results show clearer edge texture information for people and houses, with contrast more suitable for human observation, achieving the best visual effect and the highest quantitative evaluation metrics. This indicates that reasonable parameter settings can significantly enhance the quality of fusion images and quantitative evaluation metrics.

**Fig 11:**
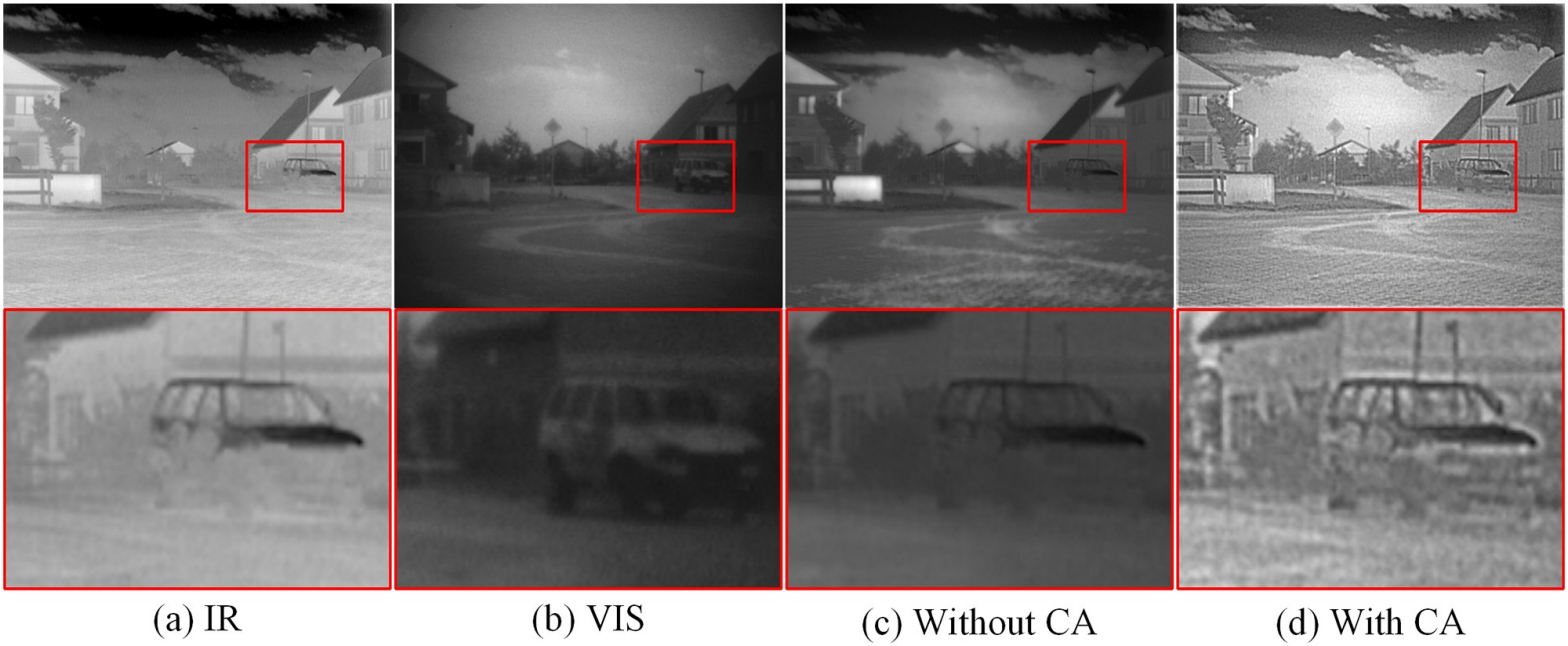
Representative results of ablation experiment with and without channel attention. (a) Infrared image, (b) Visible image, (c) Without channel attention, (d) With channel attention.

(2) Channel Attention Experiment: The utilization of the channel attention module significantly improves the performance of the model. It assists the model in automatically selecting and enhancing important input features, thereby improving the accuracy and generalization ability of the model. Additionally, the introduction of the channel attention module reduces the number of parameters in the model, thus enhancing computational efficiency. In the comparative experiments, we designed two experimental groups: one group employed the channel attention module while the other group did not. By comparing the results of these two experimental groups, we can evaluate the impact of the channel attention module on the model’s performance. The experimental results are shown in Fig 11. The results show that the generated fusion image without the channel attention module has obvious lack of contour information. The features and texture details of the car have been lost in the enlarged region, the entire image is blurred, the contrast is definitely insufficient, and the visual impression is bad.

By introducing the channel attention module, the performance of the fusion network has been significantly improved. The contrast of the fused image is enhanced, and the details are more clearly visible, leading to an overall improvement in visual effects. In Table 4, we can observe that the fusion model achieves optimal values in all six metrics after incorporating the channel attention module. These optimal values are highlighted in red and bold in the table. The results of the ablation experiments further validate this observation and are consistent with subjective evaluations.

**Table 4:** Quantitative evaluation metrics with and without channel attention. Maximum values are highlighted in bold and red.

Method	SF	EI	CO	EN	AG	VIF
Without CA	7.5628	61.5092	68.2484	6.6217	6.3589	0.7539
With CA	**8.0318**	**64.9442**	**76.0674**	**6.8467**	**6.6295**	**0.9162**

**Fig 12:**
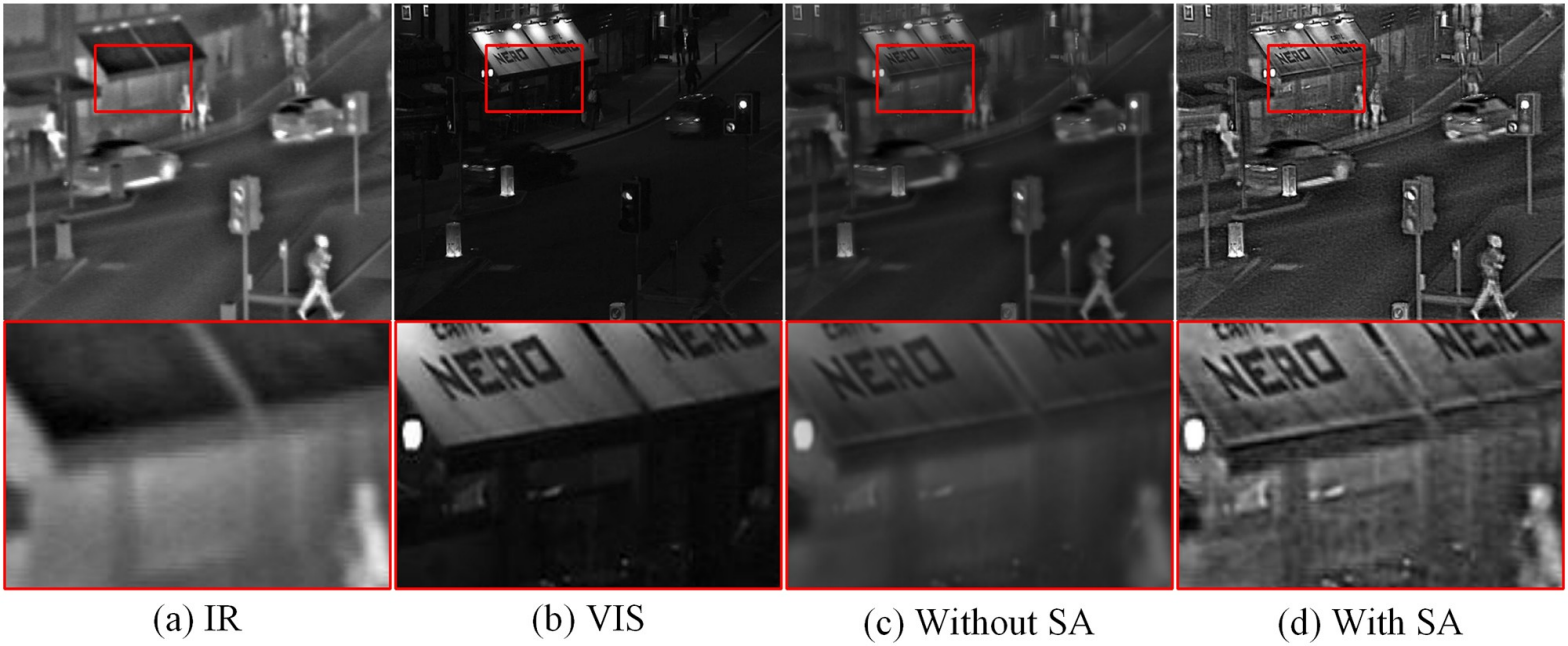
Representative results of spatial attention ablation experiment. (a) Infrared image, (b) Visible image, (c) Without spatial attention, (d) With spatial attention.

(3) Spatial attention experiment: The spatial attention module assists the model in adaptively selecting and highlighting important regions in the input data, enabling the model to better perceive and utilize crucial information. This ability to dynamically adjust the proportion of input data contributes to enhancing the accuracy and robustness of the model. We conducted comparative experiments with and without the spatial attention module to evaluate the performance difference of the model. In the magnified region highlighted in red in Fig 12, without the spatial attention module, the fusion result exhibits blurriness and loss of detail in the shop window area, resulting in poor overall visual effects. However, with the inclusion of the spatial attention module, the brightness and contrast of the fusion result are significantly improved, and the details and texture information in the shop window area are better preserved. These observations are supported by the quantitative evaluation metrics presented in Table 5, where the optimal values are highlighted in red and bold. After incorporating the spatial attention module, the fusion model achieves optimal values in these six metrics. The results of the ablation experiments further validate that the inclusion of the spatial attention module significantly enhances the performance of the fusion network.

**Table 5:** Quantitative evaluation metrics under conditions with and without spatial attention. The maximum value is highlighted in bold red.

Method	SF	EI	CO	EN	AG	VIF
Without SA	7.1639	64.3227	73.1174	6.4618	6.0826	0.8062
With SA	**8.0318**	**64.9442**	**76.0674**	**6.8467**	**6.6295**	**0.9162**

## Conclusions

This paper proposes an infrared and visible image fusion method based on a specific dual attention mechanism, aiming to obtain fused images with clear texture details and remarkable visual effects. The SDAM approach adopts an end-to-end network structure, eliminating the need for complex fusion rules and reducing the parameter and computational complexity. By incorporating both channel attention and spatial attention modules, SDAM effectively utilizes the texture details information from the visible light image and enhances the brightness and contrast of the fusion result. Additionally, an optimized composite loss function is introduced in this study, combining content loss, edge loss, and structural loss to achieve improved fusion performance. Experimental results demonstrate that compared to five state-of-the-art fusion methods, SDAM exhibits excellent performance in both subjective and objective evaluations. By leveraging the characteristics of both image sources, SDAM can generate fused images with clear texture details and optimal visual effects.

The fusion method proposed in this paper will be further optimized and deployed on embedded computing platforms in the future. Additionally, there are plans to apply the proposed fusion method in relevant fields such as medicine and remote sensing.
